# Brain Alterations in High Fat Diet Induced Obesity: Effects of Tart Cherry Seeds and Juice

**DOI:** 10.3390/nu12030623

**Published:** 2020-02-27

**Authors:** Maria Vittoria Micioni Di Bonaventura, Ilenia Martinelli, Michele Moruzzi, Emanuela Micioni Di Bonaventura, Maria Elena Giusepponi, Carlo Polidori, Giulio Lupidi, Seyed Khosrow Tayebati, Francesco Amenta, Carlo Cifani, Daniele Tomassoni

**Affiliations:** 1 School of Pharmacy, Pharmacology Unit, University of Camerino, via Madonna delle Carceri, 9, 62032 Camerino, Italy; 2 Department of Medicine, University of Leipzig, Liebigstraße 21, 04103 Leipzig, Germany; 3 School of Biosciences and Veterinary Medicine, University of Camerino, via Gentile III da Varano, 62032 Camerino, Italy

**Keywords:** Diet-Induced Obese Rats, high fat diet, brain, neuroinflammation, obesity, tart cherries

## Abstract

Evidence suggests that obesity adversely affects brain function. High body mass index, hypertension, dyslipidemia, insulin resistance, and diabetes are risk factors for increasing cognitive decline. Tart cherries (*Prunus Cerasus* L.) are rich in anthocyanins and components that modify lipid metabolism. This study evaluated the effects of tart cherries on the brain in diet-induced obese (DIO) rats. DIO rats were fed with a high-fat diet alone or in association with a tart cherry seeds powder (DS) and juice (DJS). DIO rats were compared to rats fed with a standard diet (CHOW). Food intake, body weight, fasting glycemia, insulin, cholesterol, and triglycerides were measured. Immunochemical and immunohistochemical techniques were performed. Results showed that body weight did not differ among the groups. Blood pressure and glycemia were decreased in both DS and DJS groups when compared to DIO rats. Immunochemical and immunohistochemical techniques demonstrated that in supplemented DIO rats, the glial fibrillary acid protein expression and microglial activation were reduced in both the hippocampus and in the frontal cortex, while the neurofilament was increased. Tart cherry intake modified aquaporin 4 and endothelial inflammatory markers. These findings indicate the potential role of this nutritional supplement in preventing obesity-related risk factors, especially neuroinflammation.

## 1. Introduction

Obesity is defined as the accumulation of excess body fat leading to a high body mass index (BMI > 30) [1]. Obesity is considered a dramatic global public health problem (globesity). Scientists have expected that by 2030, obesity and overweight may reach 89% in males and 85% in females, with a related increase in obesity-associated pathologies [1,2].

Excess body weight is a risk factors for several diseases, such as cardiovascular diseases, diabetes, and cancers [3,4].

Numerous experimental studies using animal models of high fat diet (HFD) induced obesity have revealed modifications in the hippocampal structure and function associated with learning and memory deficits, as well as impaired executive function [5]. It has been observed that long-term potentiation in the hippocampus, which is considered to be a major mechanism in learning and memory, is impaired in HFD-fed mouse models [6]. Moreover, HFD also leads to a reduction in markers of neurogenesis, synaptic plasticity, and neuronal growth, such as brain-derived neurotrophic factor (BDNF) [7].

Early markers of inflammation, including tumor necrosis factor-alpha (TNF-α) and microglial activation, are present in the arcuate nucleus of the hypothalamus in animal models of obesity [8]. Glial cell activation is an early essential step in HFD-induced hypothalamic inflammation, associated with reactive astrocytes and microglia [9].

Tart cherry (*Prunus Cerasus* L.), which is a rich and edible fruit, represents a source of anthocyanins and phytochemical flavonoids, which are found in red-, blue-, and purple-pigmented fruits and vegetables [10]. The components of these plants can modify lipid metabolism in vitro and reduce hyperlipidemia in vivo [11].

Cherries are highly rich in bioactive compounds and nutrients, with relatively low caloric content. These fruits are a nutritionally dense food, in which quercetin, potassium, fiber, vitamin C, carotenoids, melatonin, tryptophan, and serotonin can be found [12,13,14]. In addition to polyphenols, anthocyanins are also highly present in these fruits, mainly cyanidin-3-glucoside and cyanidine-3-rutinoside [15].

Anthocyanins, in particular cyanidin-3-glucoside, were revealed to have a great oxygen radical absorbance capacity in vitro [16], and it is well-known that the antioxidants are able to eliminate reactive oxygen species (ROS)

Several other studies demonstrated the antioxidant properties of anthocyanins in vivo. Cyanidin-3-glucoside improved oxidative stress induced by hepatic ischemia reperfusion in rats [17]. Others showed the anti-inflammatory effects of anthocyanins. The majority of antioxidants in tart cherries are present in the edible part of the fruit. High consumption of those fruits promotes health, reducing the risk for several chronic inflammatory diseases, including arthritis, cardiovascular disease (CVD), diabetes, and cancer. Moreover, cherry consumption may help improve sleep, cognitive function, and recovery from pain after strenuous exercise. Cyanidin-3-glucoside, delphinidin-3-glucoside, and petunidin-3-glucoside inhibited NF-κB activities via mitogen-activated protein kinase (MAPK) pathways [18], and cyanidins inhibited cyclooxygenase enzyme activities [19].

On the other hand, dyslipidemia, hypertension, impaired glucose tolerance, and insulin resistance often co-occur with obesity, in which adipose tissue accumulation and metabolic changes increase the incidence of heart failure and cerebrovascular disease. All these pathologies present inflammatory components that could be ameliorated with tart cherry supplementation.

Here, we investigated the potential effects of tart cherries on the brain in obese rats using a diet-induced obese (DIO) model. This preclinical model is a useful tool to study the obesity state, sharing several common features with human conditions. Indeed, the availability of HFD and the resulting overconsumption represents the etiology of obesity in modern societies. The effects of tart cherry supplementation on neuronal, glial, and inflammatory markers were evaluated on the brain of DIO rats after 17 weeks of HFD.

The expected results could contribute to clarifying (I) if the obesogenic metabolic conditions may be a promoters of neurodegenerative disorders and (II) if a diet supplemented with tart cherry seeds powder or juice may improve the brain functions in obese rats.

## 2. Materials and Methods

### 2.1. Animal Handling

Male Wistar rats (Charles River; total *n* = 44; 225–250 g, 7 weeks old at the beginning of the experiments) were used. Rats were housed in individual cage in a 12-h light/12-h dark cycle (lights on at 9:00 a.m.) with access to food and water ad libitum for 2 weeks before the experiments. They were kept in a room at constant temperature (20–22°C) and humidity (45%–55%). All procedures involving rats were carried out in accordance with the Institutional Guidelines and complied with the Italian Ministry of Health (protocol number 1610/2013) and associated guidelines from the European Communities Council Directive.

Rats were randomly divided into two groups with comparable mean body weight (no significant difference). The first group (*n* = 8) was fed with standard laboratory chow ad libitum (4RF18, Mucedola, Settimo Milanese, Italy; 2.6 kcal/g), called CHOW rats. The second group (*n* = 36) was fed ad libitum with a HFD (45% fat, 35% carbohydrate, 20% protein; D12451, Research Diets, Inc., New Brunswick, NJ; 4.73 kcal/g), called DIO rats.

DIO group was further subdivided into 3 groups: control rats (*n* = 12), rats with supplementation of *Prunus Cerasus* L. seed powder 0.1 mg/g/die (DS rats; *n* = 12), and rats with supplementation of *Prunus Cerasus* L. seed powder (0.1 mg/g/die) and juice containing 1 mg of anthocyanins (DJS rats; *n* = 12). After 5 weeks (12 weeks of age) of DIO, the obese phenotype was developed. Six of the thirty-six rats fed with HFD were excluded from the study because they did not significantly increase body weight compared to CHOW rats [20]. For 17 weeks, body weight and food intake were monitored daily, while systolic blood pressure was measured weekly.

### 2.2. Preparation of Seed Powder and Juice from Tart Cherries

Fresh tart cherries were pitted manually and mashed using a blender at room temperature for 5 min, and then with an UltraTurrax for 1 min. The homogenate was then centrifuged at 7000 x *g* for 10 min; the extract was removed and stored at 4 °C until analyzed. The precipitate was further extracted in 96% ethanol for one night. The solution was centrifuged at 10,000 x *g* for 20 min and the supernatant (ethanol extract) was collected and evaporated using a rotary evaporator. The concentrated juice was added to the pulp extract and standardized so that the rats could be given 1 mg of anthocyanins every day. Juice was given inside a standard water bottle. The total monomeric anthocyanin content was measured by the differential method [21]. The dried seeds, separated from the shells, were ground and degreased with two ultrasound extraction rounds using 30 mL of petroleum ether. These seeds were incorporated into the standard diet and administered to each animal at 0.1 mg/g per day [22,23] for 17 weeks. For the DIO rats, 0.1 mg/g of seed powder was added to 1 g of animal fat.

### 2.3. Behavioral Tests

Two weeks before sacrifice, a battery of behavioral and memory tests was performed to detect potential behavioral abnormalities, in the following specific order, to avoid potential between-test interference effects: open field test, passive avoidance test, and Morris water maze.

#### 2.3.1. Open Field test

Automated locomotor activity boxes (square plastic boxes with a 43 × 43 cm arena and a 25 × 25 cm virtual central zone; Med Associates, St Albans, Vermont, USA) were used to quantify spontaneous locomotor activity parameters. Activity was recorded for 10 min, starting 1 min after the placement of the animal in the test cage. Locomotor activity was recorded automatically by interruption of two orthogonal light beams (3.5 and 13 cm above the activity box floor), which were connected to automatic software. Locomotion counts were recorded when the low row of photocells was interrupted. Locomotion in the entire open field and in the central zone and entries into the central zone were computed based on interruptions of infrared light beams placed in a 2.6 × 3 × 2.6 cm orthogonal grid situated 3.5 cm above the box floor. Increased locomotor activity in the entire field was considered a sign of behavioral arousal; and reduced locomotor activity in the central zone and numbers of entries into the central zone were considered signs of increased emotionality, anxiety, or fear in mice and rats [24,25]. Between test sessions, the apparatus was cleaned with alcohol (70%) and dried with a cloth.

#### 2.3.2. Passive avoidance test

A passive avoidance learning test was performed according to Eagle and collaborators (2016) [26], using an apparatus composed of light and dark chambers of identical size (20 cm × 20 cm × 40 cm) with a rectangular door in the middle (8 cm × 8 cm) connecting the two chambers. Briefly, the animals were placed into the light side of the box and the time taken to cross into the dark side was measured. During the passive avoidance training, after entry into the dark chamber, the animals received a mild (1.24 mA 3 s) foot shock and were then removed from the box. Electric shock was delivered to the grid floor by a constant current generator (Med Associates). For the retention assessment, after 72 h the animal was placed back into the box and cross-over latency was measured, up to a maximum of 300 s as the cut-off.

#### 2.3.3. Morris water maze

The apparatus consisted of a circular pool (diameter 200 cm, height 60 cm) located in a test room with several cues on the walls. The inner surface of the pool was painted black, filled to a depth of 40 cm with water (maintained at 24 ± 1°C), which covered an invisible (black) 10-cm square platform. The platform was submerged approximately 1 cm below the surface of the water. The pool was virtually divided into 4 quadrants and the platform was placed in a fixed position at the center of one of the four quadrants of the maze. Rats underwent 4 trial training sessions separated by 24 h for 4 consecutive days. Briefly, for each trial, rats were allowed to swim until they reached the escape platform. A cut-off of 60 s was chosen, at the end of which the animal was placed on the platform where it was left for a reinforcement period of 30 s. The starting position of the animal randomly varied among four equally spaced positions around the perimeter of the pool. Spatial learning was assessed across repeated trials, measuring the time rats took to reach the platform (escape latency) [27]. Latency to escape is the primary parameter of an animals’ cognitive ability, and a shorter latency time indicates better performance [28]. After the last day trial, the animal received a 60 s probe test, in which the platform was removed from the pool and the time taken to reach the platform quadrant, which previously contained the platform during the training, and swim speed were measured [29].

### 2.4. Blood and Brain Tissue Sampling

Before sacrifice and after systolic blood pressure measurement, blood withdrawals were performed from the tail vein. In total, 800 µl of blood was collected in tubes with L-heparin. The blood samples were then centrifuged for 10 min at 3000 rpm. Blood parameters (glucose, total cholesterol, and triglycerides) were evaluated by IDEXX Catalyst Dx. Insulin was determined using an ^®^Ultrasensitive Rat Insulin ELISA (EIA-2943 DRG Diagnostic, Germany) kit. After sacrifice, the brains were removed and immediately frozen for biochemical analysis or immersed in 4% paraformaldehyde solution 0.1 M Phosphate Buffer Saline (PBS) at pH 7.4 for 48 h for paraffin embedding. After fixation, the samples were gradually dehydrated in ethanol using crescent solution with concentrations ranging from 70% to 100%. Once successfully dehydrated, xylene was infiltrated through the tissues. Finally, the samples were embedded in paraffin through successive steps from 42 to 60°C (1 h for each step). After embedding, the brain samples were cut using the microtome into sections and processed for immunohistochemistry (IHC) analysis.

### 2.5. Western Blot Analysis

Samples of brain (0.1 ± 0.02 g) were homogenized in lysis buffer containing protease inhibitor cocktail (Sigma-Aldrich). The supernatant was used for protein assay against a standard of bovine serum albumin (BSA) using a Bio-Rad Protein Assay [catalogue number (Cat. No.) 500–0001, Bio-Rad Laboratories, Inc., Munich, Germany)]. Next, 40 μg of proteins was separated using 8%–14% Sodium Dodecyl Sulfate (SDS) polyacrylamide gel and transferred onto nitrocellulose membranes. The nitrocellulose membranes were incubated with the following antibodies (Table 1): neuronal nuclei protein (Neu-N), neurofilament 200 KDa (NF), glial fibrillary acidic protein (GFAP), ionized calcium-binding adapter molecule 1 (IBA-1), aquaporin 4 (AQP4), intercellular adhesion molecule 1 (ICAM-1), and vascular cell adhesion molecule 1 (VCAM-1). The blots were washed with PBS containing 0.5% Tween 20 (PBS-T) and then incubated with the horseradish-peroxidase-linked secondary antibodies (goat anti-rabbit IgG, Cat. No. A120–101P; or goat anti-mouse IgG, Cat. No. A90–116P, Bethyl Laboratory, Inc., Montgomery, TX, USA) at 1:5000 dilution for 60 min at room temperature, followed by visualization with an enhanced chemiluminescence kit (Lite Ablot^®^ plus, Cat. No. EMP 011005, Euroclone, Life Sciences Division, Siziano, Italy). The images were acquired by the ChemiDoc imaging system (Bio-Rad Laboratories, Inc.). The β-actin protein (clone AC-74, Cat. No. A2228, Sigma-Aldrich Co., St. Louis, MO, USA) was used as a loading control at 1:3000 dilution in PBS-T overnight at 4°C. Band intensities were measured by densitometry with Nikon Imaging Software (NIS Elements) (Nikon, Florence, Italy). Blots are representative of three different experimental sessions.

### 2.6. Histochemistry and Immunohistochemistry

Serial sagittal consecutive 10-μm thick sections containing frontal cortex (including layers I–IV and layers V–VI, along with corresponding white matter) and hippocampus (lateral 1.40 - 1.90 mm) samples [30] were stained with Nissl’s method (cresyl violet 1.5%) for morphometric analysis and to assess the occurrence of relevant microanatomical changes. Alternative brain sections were exposed to different antibodies (Table 1) diluted in 1X PBS/0.3% Triton X-100 (PBS/Triton) (200 μL per section). Slides were incubated overnight at 4 °C with primary antibodies (Table 1) and the product of the immune reaction was then revealed by incubating slides for 30 min at 25°C with the specific biotinylated secondary IgGs (goat anti-rabbit IgG, Cat. No. A120–101B; or goat anti-mouse IgG, Cat. No. A90–116B, Bethyl Laboratory, Inc., Montgomery, TX, USA) at a dilution of 1:200 in PBS/Triton. The immune reaction was then revealed with diaminobenzidine (0.05% 3, 3'–-diaminobenzidine dissolved in 0.1% H_2_O_2_) as a substrate. Slides were then washed, mounted on cover slips, and viewed under a light microscope. Before dehydration in ethanol, sections were also counterstained with hematoxylin. Some sections were incubated with a non-immune serum instead of a primary antibody to assess the immunostaining background.

### 2.7. Morphometric Analysis

After mounting, sections were analyzed in the frontal cortex, the CA1 or CA3 hippocampal areas, and the dentate gyrus. The analysis was done with a computer-assisted, semi-quantitative method; images were recorded on a light microscope using a camera installed on the microscope itself and translated into an image analysis software, as previously described [31]. The NIS (Nikon) software is designed for quantitative analysis. It was used on the brain sections, which were tested for Neu-N to assess the number of neurons per area. Moreover, the sections immunostained with the antibody to GFAP were used to determine the occurrence of hypertrophy. Images were acquired with 20x magnification in the Neu-N stained slides and at 40x magnification for the GFAP-stained slides. For NF and AQP4, the mean intensity of immunoreaction was measured with NIS Elements (Nikon) software options, with 0 (white) representing the total absence of immunoreaction of the negative control and 256 (black) being the highest value of immunoreaction [31].

### 2.8. Statistical Analysis

In vivo and ex vivo data are expressed as mean ± standard error of the mean (S.E.M), and were analyzed by one-way analysis of variance (ANOVA) and time as the within-subject variables when appropriate, followed by post hoc comparison carried out by the Bonferroni test. The *p*-values < 0.05 were considered to be statistically significant. The data were analyzed with analysis of variance (ANOVA) (Systat Software 10.0). 

## 3. Results

### 3.1. Body Weight, Food Intake, and Blood Parameters

As expected, we found that body weight and food intake were increased in DIO rats in comparison to CHOW rats (Table 2). The tart cherry seed and juice supplementation did not affect body weight or feeding behavior (Table 2).

Systolic blood pressure was higher in DIO rats after 17 weeks of HFD compared to age-matched CHOW rats. DS and DJS rats showed a significant reduction of systolic blood pressure compared to DIO animals (Table 2).

The obese condition induced an increase of glucose and insulin levels in the DIO rats after 17 weeks of HFD. Tart cherry intake reduced only the hyperglycemia but not the hyperinsulinemia (Table 2). Obesity did not significantly affect total cholesterol and triglycerides levels (Table 2). However, the tart cherry intake remarkably reduced the triglyceride concentration compared to the DIO rats (Table 2).

### 3.2. Behavioral tests

#### 3.2.1. Open Field test

After 15 weeks of HFD, the entire arena of the locomotor activity (*p* < 0.01), the other behavioral parameters (vertical (*p* < 0.05), and jump (*p* < 0.01) count) were significantly decreased in DIO rats compared to CHOW rats. These results were expected due to the significant increase in body weight in DIO rats (Table 3). Interestingly, DIO rats showed anxiogenic behavior; indeed, the zone entries (*p* < 0.05) and the distance travel (*p* < 0.01) in the center of the arena were significantly reduced compared to lean rats (Table 3).

DIO rats with supplementation did not differ in their performance in the open field, traveling the same distance (cm/10 min) in the entire arena or in the center of the arena. 

#### 3.2.2. Passive avoidance test

The passive avoidance test (Table 3) revealed a significant difference in the latency time (expressed in seconds, s) among the groups (*p* < 0.05). Specifically, the latency time in DIO groups was significantly less than the CHOW group (*p* < 0.05). The supplementation in DIO rats did not affect the impairment of learning caused by obese state.

#### 3.2.3. Morris water maze

**Figure 1:**
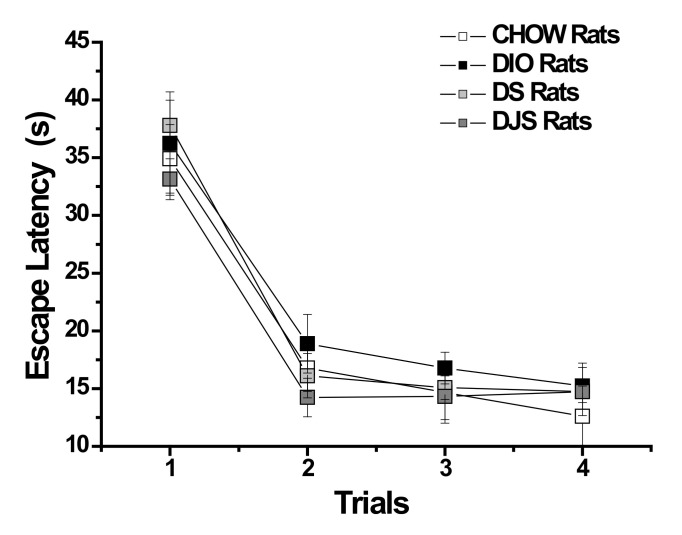
Morris water maze. Each trial shows the average (four trials each day) of the escape latency time taken to reach the platform on all 4 days of the test. Data presented as mean ± S.E.M.

Figure 1 shows the learning progression during 4 days of training (four trials each day). Each trial represented the escape latency time taken to reach the platform in the same day trial. DIO rats took more time to reach the platform; however, this trend did not reach statistical significance (*p* > 0.05). In Table 3, we summarized the findings measured during the probe test. DIO rats significantly increased the time (s) taken to reach the platform quadrant (*p* < 0.05), while they decreased the time taken in the target quadrant (*p* < 0.05) and the swim speed (*p* < 0.05) tests. The supplementation in DIO rats did not affect the memory task (Table 3).

### 3.3. Neuronal Markers

**Figure 2:**
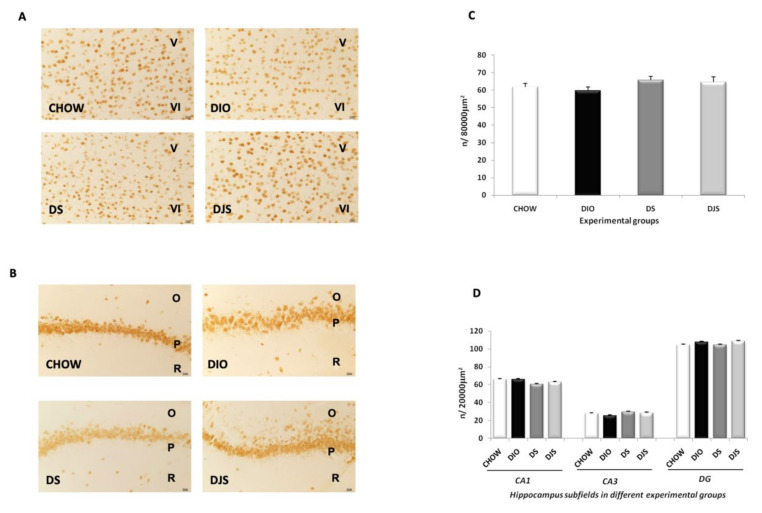
Neuronal nuclei protein expression in the different brain areas. Sections of the frontal cortex, V-VI layers (**A**), and the CA1 subfield of the hippocampus (**B**) were processed for Neu-N immunohistochemistry in the different experimental groups. O, *stratum oriens*; P, pyramidal neurons; R, *stratum radiatum*. Calibration bar: 25 μm. Morphometric analysis of a number of pyramidal neurons in the fifth layer of the frontal cortex (**C**) and the different subfields of the hippocampus (**D**) was performed in sections processed for Neu-N immunohistochemistry. Data are the mean ± S.E.M.

The sections processed for Neu-N immunohistochemistry showed a clear expression of this protein in the nucleus of the neurons in the frontal cortex (fifth and sixth layers) (Figure 2A) and in the pyramidal neurons of the hippocampal CA1 subfield (Figure 2B). Morphological analysis revealed no difference in the number of immunoreactive neurons among the different experimental groups, in either the fifth layer of the frontal cortex (Figure 2C) or in different subfields of the hippocampus (CA1, CA3, and dentate gyrus) (Figure 2D).

**Figure 3:**
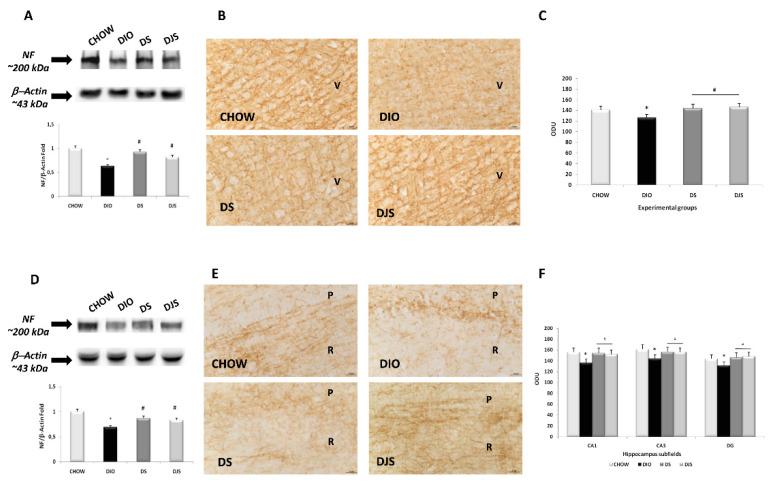
Expression of neurofilament in the different brain areas. Western blot analysis (representative of three different experimental sessions) for samples of the frontal cortex (**A**) and hippocampus (**D**) were probed for NF and β-actin, with corresponding densitometric analysis of the bands. Sections of the frontal cortex (**B**) and CA1 subfield of the hippocampus (**E**) were processed for neurofilament immunohistochemistry, with densitometric analysis of the immunoreaction intensity for the frontal cortex (**C**) and hippocampus (**F**). V, the fifth layer of the frontal cortex; P, pyramidal neurons; R, *stratum radiatum* of the hippocampus. The values are the mean ± S.E.M.; * *p* < 0.05 vs. CHOW rats; ^#^ *p* < 0.05 vs. DIO rats. Calibration bar: 25 μm.

Immunoblots of brain areas against the high molecular weight NF revealed a band at approximately 200 kDa (Figure 3A,D). The migration patterns of the immunoreactions were similar in the four animal groups investigated. The intensity of the bands, normalized for the corresponding β-actin expression, decreased in both the frontal cortex (Figure 3A) and in the hippocampus of DIO rats compared to CHOW group (Figure 3D). In the DS and DJS, the expression increased in comparison to the DIO rats (Figure 3A,D). Sections processed for NF immunohistochemistry developed dark-brown staining along the axons of frontal cortex and hippocampal CA1 subfield (Figure 3B,E, respectively), as in the CA3 subfield and dentate gyrus (data not shown). The density values of immunoreactivity for NF in the frontal cortex, in the CA1 and CA3 subfields of the hippocampus, and in the dentate gyrus significantly decreased in the DIO rats compared to the CHOW group (*p* < 0.05) (Figure 3C,F). However, densitometry revealed an intensification of the intensity values in DS and DJS compared to the DIO rats (*p* < 0.05) (Figure 3C,F), indicating a higher NF expression with tart cherry intake.

### 3.4. Glial Markers

**Figure 4:**
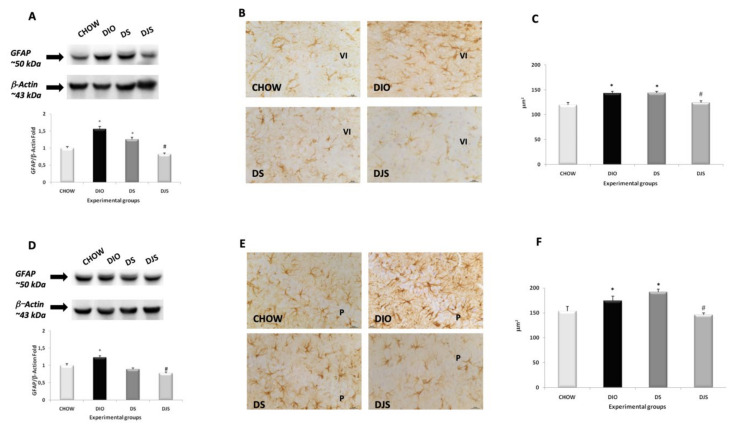
Expression of glial fibrillary acidic protein (GFAP). Western blot analysis for samples (representative of three different experimental sessions) of the frontal cortex (**A**) and hippocampus (**D**) probed for GFAP and β-Actin, with the corresponding densitometric analysis of the bands. Sections of the frontal cortex (**B**) and CA1 subfield of the hippocampus (**E**) processed for glial fibrillary acidic protein and the morphological analysis of the mean area of immunoreaction for the frontal cortex (**C**) and hippocampus (**F**). VI, sixth layer of the frontal cortex; P: pyramidal neurons. The values are the mean ± S.E.M.; ∗*p* <0.05 vs. CHOW rats; #*p* < 0.05 vs. DIO rats. Calibration bar: 25 μm.

For GFAP immunoblots, a single band of approximately 50 kDa (Figure 4A,D) was revealed in the frontal cortex and hippocampus of the different groups. The intensity of the GFAP bands, normalized for the corresponding reference protein expression, was increased in both brain areas of DIO rats compared to CHOW rats (Figure 4A,D). Moreover, decreased expression of GFAP in DJS group compared to DIO was detected in both the frontal cortex and hippocampus (Figure 4A,D). The data from the immunohistochemistry analysis were consistent with Western blot results. In the frontal cortex and in the CA1 subfield, a marked increase in GFAP immunoreactive astrocytes was evident in the DIO group in comparison with the control, as well as a decrease in the DJS group (Figure 4 B,C,E,F), which seems to be the most effective treatment. In the sixth layer close to the corpus callosum, the size of astrocytes was larger in DIO rats than in the CHOW rats, whereas the tart cherries reduced the size (*p* < 0.05) (Figure 4B,C). In the hippocampus, in particular in the CA1 subfield, an increase of the mean immunoreactions area of GFAP hyper-reactive astrocytes was evident in rats fed with HFD (Figure 4E,F). Moreover, the groups of animal supplemented with tart cherry juice showed a significant decrease in the mean values of the immunoreaction area (*p* < 0.05) (Figure 4E,F).

**Figure 5:**
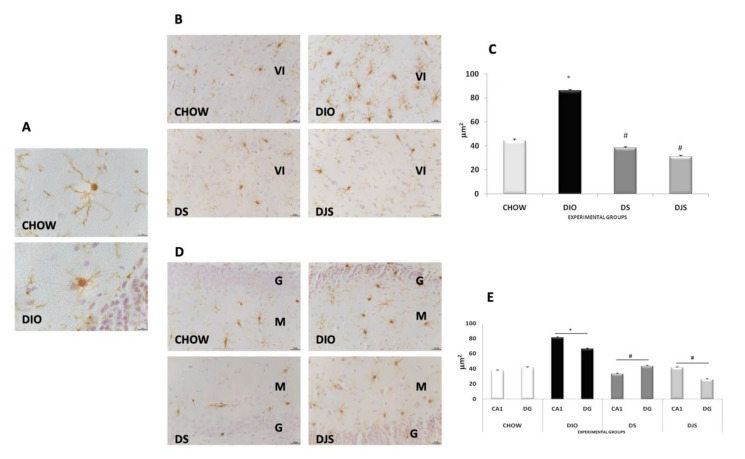
Microglial reaction. Immunohistochemical analysis of microglial IBA-1-positive cells in the frontal cortex (**A** and **B**) and dentate gyrus (**D**). In DIO rats, an increase of the soma and a reduction of arborizations of glial cells were noticeable (A). Morphometric analysis of the area of the soma in the different experimental groups in the frontal cortex (**C**) and hippocampus (**E**). VI, sixth layer of the frontal cortex; G, granular layer of the dentate gyrus; M, molecular layer of the dentate gyrus. The values are the mean ± S.E.M.;∗*p* < 0.05 vs. CHOW rats; #*p* < 0.05 vs. DIO rats. Calibration bar: panel A: 10 μm; panels B and D: 25 μm.

Sections processed for immunohistochemistry against ionized calcium-binding adapter molecule 1 (IBA-1), as markers of microglia cells, revealed an increase of activated microglia cells in DIO rats compared to the controls (Figure 5). Obesity induced a morphological change in microglia in brain areas. In CHOW rats, resting microglia was present at the level of different layers of the frontal cortex, in the *stratum radiatum* of the CA1 subfield, and in the hilum and *stratum moleculare* of the dentate gyrus (Figure 5B,D). In obese rats, typical elements of the reactive microglial cells were evident, with a clear increase in the area of soma without changes in the arborizations, as demonstrated in Figure 5A, obtained at 100x magnification. No phagocytic microglia were present in the frontal cortex or in the hippocampus of obese rats. Morphometric analysis highlighted a clear increase of the area of the soma in the DIO rats compared to the lean CHOW rats (*p* < 0.05) (Figure 5C,E). In the sections of DS and DJS groups, a decrease of the immunoreactive microglial cells was evident, with a significant decrease (*p* < 0.05) of the area of the soma (Figure 5B–E).

**Figure 6:**
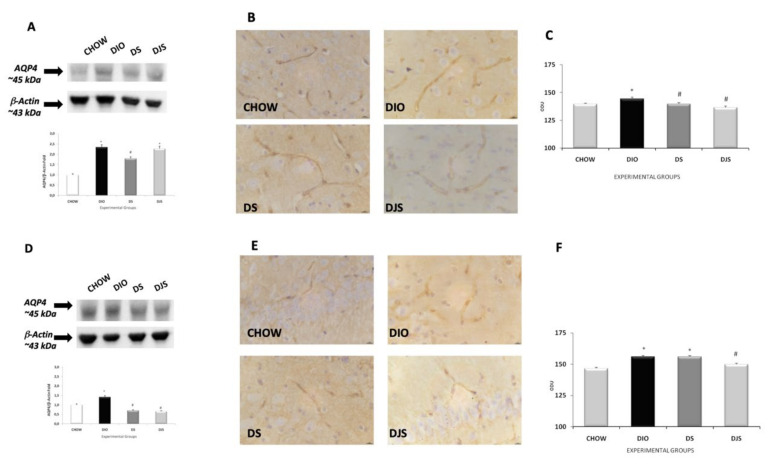
Expression of aquaporin 4 (AQP4) as a blood–brain barrier marker. Western blot analysis (representative of three different experimental sessions) for samples of the frontal cortex (**A**) and hippocampus (**D**) probed for AQP4 and β-Actin, with the corresponding densitometric analysis of the bands. Sections of the frontal cortex (**B**) and CA1 subfield of the hippocampus (**E**) processed for AQP4 immunohistochemistry and the corresponding morphological analysis of the mean area of immunoreaction for the frontal cortex (**C**) and hippocampus (**F**). The values are the mean ± S.E.M.; ∗*p* < 0.05 vs. CHOW rats; #*p* < 0.05 vs. DIO rats. Calibration bar: 25 μm.

Exposure of membranes of the frontal cortex and hippocampus to anti-AQP4 antibody caused the development of a band at approximately 45 KDa (Figure 6 A,D) The results showed an increase of the AQP4 in the frontal cortex of the DIO compared to the CHOW rats (Figure 6A). In the frontal cortex, only in the DS group was there a decrease of AQP4 compared to the DIO rats. In the hippocampus of the DS and DJS groups, the AQP4 expression was reduced compared to the age-matched DIO rats (Figure 6A,D). Sections processed for AQP4 immunohistochemistry developed dark-brown staining around brain microvessels. The AQP4 expression was higher in DIO rats (Figure 6B,C) than CHOW rats and decreased in the DS and DJS groups (*p* < 0.05) (Figure 6B,C). At the level of the hippocampus, the densitometry analysis showed an increase of AQP4 immunoreactivity in DIO compare to CHOW rats (Figure 6E,F). On the contrary, a decrease of AQP4 was evident in the animals supplemented with tart cherry seeds and juice compared to the DIO rats (Figure 6F).

### 3.5. Endothelial Inflammatory Markers

**Figure 7:**
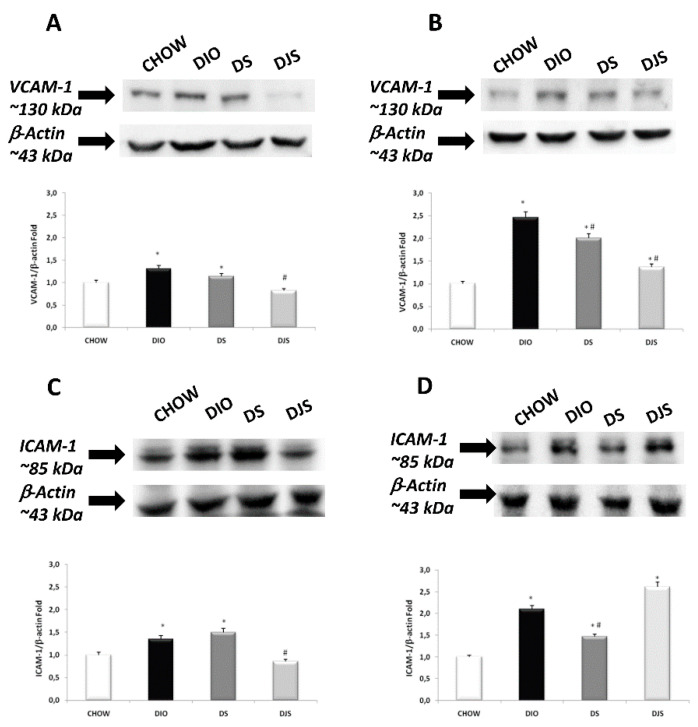
Western blot analysis (representative of three different experimental sessions) for VCAM-1 in the frontal cortex (**A**) and hippocampus (**B**), and for ICAM-1 in the frontal cortex (**C**) and hippocampus (**D**) in the CHOW, DIO, DIO after supplementation with tart cherry seeds (DS), and DIO after supplementation with seeds and tart cherry juice (DJS) groups. Graphs represent the intensity of bands normalized to the band intensity of the reference protein β-actin. The values are the mean ± S.E.M.; * *p* < 0.05 vs. CHOW rats; ^#^ *p* < 0.05 vs. DIO rats.

Exposure of membranes to anti-VCAM-1 antibodies caused the development of a 130 kDa band. The expression is more evident in the frontal cortex than in the hippocampus. The presence of VCAM-1 is higher in the DIO rats compared to the CHOW ones, especially in the hippocampus. Considering the VCAM-1 intensity in the DIO rats, intake of seeds only slightly reduced its expression compared to combined supplementation with juice. This result is strongly evident in the hippocampus of the DJS group (Figure 7). For ICAM-1, a band of 85 kDa was shown in the frontal cortex and hippocampus of the different experimental groups. The intensities of the bands, normalized for the corresponding reference protein expressions, increased in DIO rats compared to CHOW rats, especially in the hippocampus. A reduction was seen in the frontal cortex of the DJS group and in the hippocampus of the DS group (Figure 7).

## 4. Discussion

A caloric-dense diet, increased food intake, reduced physical activity, and altered metabolism are the variables that affect energy balance, leading to excess weight gain and obesity [32]. Obesity is considered a medical challenge because it is associated with the progress of some chronic diseases. Despite the well-known global impact of overweight and obesity on the frequency of cerebrovascular disease and cognitive decline, many aspects of this association are still inconsistently defined [33]. Obesity is a risk factor if accompanied by hypertension, hyperlipidemia, and impaired glucose tolerance. In addition, abdominal obesity and higher BMI have roles in the development of cerebral infarction [34]. Furthermore, hypertension associated with obesity, the overconsumption of high-energy foods, and caloric or high fructose intake negatively impact brain function, inducing lower cognitive performance [35,36] or increasing the risk of dementia (Alzheimer’s disease, AD) [37,38], especially in mid-life [39,40]. Moreover, it is known that glycemic extremes (hyper- and hypoglycemia) affect brain development [41].

Experimental data in AD mouse models have established that diet-induced obesity remarkably aggravates AD-like neuropathology and worsens cognitive impairment [42,43]. Indeed, HFD induces tau protein accumulation, processing, and hyperphosphorylation [42,44], and causes mild metabolic dysfunction and cognitive impartment [43]. In non-transgenic rodents, the exposure to HFD decreases cognitive performance, leading to impaired spatial memory [45,46]. In our open field, passive avoidance, and Morris water maze test studies, DIO rats developed an increase of anxiety status and alterations in terms of learning and memory tasks. These findings suggest that the chronic consumption of a HFD increases anxiety-like behavior, as shown by other authors [47,48,49]. The cognitive impairment found in DIO rats could be related to metabolic disturbances (i.e., inflammation and insulin resistance) [50] due to cerebrovascular dysfunction, characterized by increased blood–brain barrier (BBB) permeability that allows macrophage or cytokine entry and reduced transport of trophic factors [38]. Regarding the blood parameters investigated, the levels of insulin and glycemia were higher in DIO rats compared to the controls, indicating a condition of insulin resistance typical of type 2 diabetes mellitus. Additionally, triglycerides and total cholesterol levels were similar to the CHOW group. This suggests that in order to establish a condition of hyperlipidemia, a diet with higher caloric intake or a longer period of treatment is necessary. Additionally, most wild-type mice are generally resistant to hypercholesterolemia and atherogenesis, even when fed a HFD [51]. In our study, DIO rats showed higher levels of blood pressure compared to CHOW rats. Hypertension is strictly related to the development of cerebrovascular disease [52]. Our hypertensive DIO rats showed brain alterations characteristic of those observed by other researchers in the same conditions.

Although the numbers of Neu-N positive neurons were similar between CHOW and DIO rats in the frontal cortex and hippocampus, the expression of NF changed in our obesity conditions. Indeed, our data showed a lower expression of NF in the frontal cortex and hippocampus of DIO rats, supporting the hypothesis proposed by previous authors for neurodegeneration occurrence in rats exposed to obesity-inducing diets [50]. Indeed, as reported by the international literature, the NF 200-kDa protein is a specific component of the neuronal cytoskeleton that is located in axons of pyramidal neurons of the hippocampus and frontal cortex. Therefore, it could represent a good marker of axonal damage [53]. The loss of NF immunoreactivity in the CA1 subfield of the hippocampus of DIO rats could suggest damage to, or breakdown of, the cytoskeleton rather than an axonal decline consequent to nerve cell loss. A decrease of NF immunoreactivity was reported to be more pronounced than the nerve cell loss [54]. It is possible to speculate that the pronounced loss of phosphorylated NF protein immunoreactivity maybe have been caused by changes to the neuronal environment in obesity, inducing cytoskeletal breakdown insufficient to cause neuronal death. Cytoskeletal breakdown has been reported to occur in several central and peripheral neurodegenerative diseases, and it may increase neuronal susceptibility to ischemia or hypoxia [53,55], two situations more common in obesity than in normal conditions.

GFAP overexpression in astrocytes was recently described in obese animals [56]. Our findings are similar. This astrogliosis suggests that the neuroinflammatory response also occurs in the hippocampus [56]. Additionally, microglia activation has been reported to be associated with the obesity phenotype [57,58], as shown by the microglia morphology modifications stained for IBA-1 at the level of the frontal cortex and hippocampus.

The nervous system cellular environment is modulated by microglia activity, which can induce neuroprotective and neurotoxic effects. Especially in response to pathological conditions, microglia produce free radicals and pro-inflammatory cytokines, molecules that can contribute to axon demyelination and neuron death. For this reason, activation of microglia functions promotes defense and repair mechanisms, as well as improves brain injuries, such as progressive neurodegenerative disorders [59].

It has been suggested that AQP4 plays an important role in BBB function and in the pathogenesis of hypertensive cerebral injury [60,61]. In spite of the above data, the role of AQP4 in maintaining BBB integrity has not yet been clarified. AQP4 is probably involved in brain edema formation and resolution, although its exact role in edema pathophysiology is unclear [62]. AQP4 may increase in edema, and its deletion protects mice brain from edema [63]. AQP4 overexpression has also been found to decrease cytotoxic edema in a hypoxia–ischemia rat model [64]. Our results showed an increase of AQP4 in cerebral areas of DIO rats, similar to increases observed in animal models of hypertension [60,61,65].

It is well known that obesity is characterized by a low grade of chronic inflammation. Inflammatory adhesion molecules, expressed by the endothelial cells, are involved in the pathogenesis of obesity, hypertension, and cerebrovascular events. The expression of VCAM-1 and ICAM-1 protein are up-regulated in response to inflammatory insult [66,67,68]. In particular, increased ICAM-1 further stimulates adhesion between vascular endothelial cells and leukocytes, damages vascular endothelial cells, and increases the permeability of capillaries, consequently inducing brain damage [69].

In this context, the potential benefit effects of tart cherry supplementation in the brains of animals fed with a HFD was evaluated.

The seed plus juice supplementation was studied to evaluate a possible improvement of the tart cherry juice performance, which was previously investigated alone [70]. Moreover, two supplements were chosen because of their different possible active compounds (flavonoids and fatty acids), which may be effective in different ways. 

Recently, it has been described that daily tart cherry juice consumption may improve cognitive abilities [70]. In our results, however, we did not observe these phenomena. Although tart cherry seeds and juice did not affect body weight in DIO rats [71], suggesting no effect on adipocyte accumulation, both the systolic blood pressure and glycaemia values in our study were reduced, as previously reported [70]. 

The effects of anthocyanin supplementation on the body weight remain controversial [72,73]. However, our data confirmed that anthocyanins are health-promoting bioactive compounds [74]. Several studies have shown that phytocompounds found in fruits and vegetables have beneficial health effects, such as prevention of cancer, cardiovascular diseases, and obesity [75]. Our findings agree with the report that tart cherry fruit showed significant blood-sugar-lowering effects [76]. 

It has been reported that the anthocyanins may exert many beneficial effects in the central nervous system [77], such as ameliorating signal transduction and neuronal communication, increasing hippocampal plasticity [78]. Moreover, Andres-Lacueva et al. [79] revealed that the presence of anthocyanins can raise the neuronal signaling pathways that are associated with memory. Furthermore, anthocyanins can reduce the development of neurodegenerative diseases by preserving normal neuronal functions and inhibiting amyloid–β peptide aggregation [77].

In line with the above studies, we showed that the density and pattern of NF in the hippocampus and frontal cortex increased significantly in DS and DJS groups compared to the DIO group. Thus, the tart cherry intake was able to positively modulate the NF expression, reducing axonal damage. Moreover, data obtained for the glial and microglial markers showed a reduction of astrocyte size in the treated groups compared to DIO. This indicates a regression of the inflammatory process, as previously demonstrated [80], in particular with juice supplementation. Our results have also shown a significant regression of the macrophagic state of IBA-1-positive cells, especially in the CA1 subfield, where we found a reduction of the activated status of microglial cells in DS and DJS groups with respect to the obese group. 

Finally, supplementation with tart cherry seeds or juice decreased the expression of some endothelial inflammatory markers. This positive effect could be related to potential vasoactive and vasoprotective properties of anthocyanin on the endothelium-dependent relaxation capacity, as suggested by other authors [81], but this was not measured in our study. Indeed, it has been reported that the nitric oxide (NO) system may be involved in the relaxation response of coronary arteries to red fruit extracts [81]. 

The main components of tart cherry seeds are oleic and linoleic acids, which have been shown to protect the endothelium [82]. These compounds could partially explain the same anti-inflammatory effects observed in the brain of DIO rats. A diet high in oleic acid can have a beneficial effect on type 2 diabetes, and can ultimately reverse the negative effects of inflammatory cytokines observed in obesity and non-insulin-dependent diabetes mellitus [83]. Oleic acid has been suggested to protect against cardiovascular insulin resistance, improving endothelial dysfunction in response to proinflammatory signals, and finally reducing proliferation and apoptosis in vascular smooth muscle cells. These activities may contribute to an ameliorated atherosclerotic process and plaque stability [84].

## 5. Conclusions

The identification of neurodegenerative changes in DIO rats may represent the first insight with which to better characterize the cerebral modifications occurring in obesity. The availability of suitable animal models may be useful for investigating nutraceutical efficacy and countering obesity-induced brain injury. Furthermore, these results may represent the first step toward clarifying the possible use of tart cherry supplementation to prevent obesity alterations in the brain. However, further studies are needed to better clarify the specific mechanisms of action of tart cherry juice and seed components in order to identify the activity of anthocyanin-rich juice or fatty-acid-rich seed powder under HFD or after alimentary lifestyle changes [85]. The results could be useful for obtaining a possible nutraceutical complex to use in cognitive dysfunction or to prevent obesity-related end organ damage.

## Figures and Tables

**Table 1:** The primary antibodies used for Western blot (WB) and immunohistochemistry (IHC) analyses.

Primary Antibody	Host Animal	Company	Dilution for WB	Dilution for IHC
Neuron-specific nuclear protein (Neu-N)	Mouse	Merk-Millipore, USA	1:1000	1:500
Neurofilament 200kDa (NF)	Mouse	Merk-Millipore, USA	1:1000	1:500
Glial fibrillary acidic protein (GFAP)	Mouse	Merk-Millipore, USA	1:1000	1:500
Ionized calcium-binding adaptor molecule 1 (IBA-1)	Mouse	Thermofisher, USA	/	1:200
Aquaporin 4 (AQP4)	Rabbit	Merk-Millipore, USA	1:500	1:500
Intercellular adhesion molecule 1 (ICAM-1)	Rabbit	Santa Cruz Biotechnology, USA	1:500	
Vascular cell adhesion molecule 1 (VCAM-1)	Rabbit	Santa Cruz Biotechnology, USA	1:500	

**Table 2:** Summary of rat parameters on the final day of the experiment.

	CHOW	DIO	DS	DJS
Body Weight (g)	557.0 ± 10.7	682.8 ± 17**	683.1 ± 29.7**	689 ± 20.8**
Food Intake (kcal)	75.7 ± 2.3	93.0 ± 3.3*	91.6 ± 7*	88.0 ± 2.6*
Systolic blood pressure (mm/Hg)	110.9 ± 6.1	140.3 ± 8.1*	111.4 ± 5.5^#^	107.6 ± 6.01^#^
Glycemia (mg/dl)	91.6 ± 5.1	126.8 ± 6.1*	105.7 ± 3.6^#^	111.3 ± 2.4*^#^
Insulin (*µ*g/L)	0.73 ± 0.05	1.06 ± 0.05*	1.01 ± 0.06*	1.03 ± 0.06*
Cholesterol (mg/dl)	76.1 ± 3.3	75.6 ± 4.1	69.8 ± 5.6	77.6 ± 4.4
Triglycerides (mg/dl)	76.6 ± 10.4	84.3 ± 13.9	42.9 ± 3.6*^#^	49.8 ± 1.9*^#^

CHOW, rats fed with a standard diet; DIO, diet-induced obese rats; DS, DIO rats fed tart cherry seeds powder; DJS, DIO rats fed tart cherry seeds powder and cherry juice. Data are the mean ± S.E.M; *p < 0.05, **p < 0.01 vs. CHOW rats; ^#^*p* < 0.05 vs. DIO rats.

**Table 3:** Behavioral tests.

OPEN FIELD TEST				
	CHOW	DIO	DS	DJS
Total distance travel (cm/10 min)	4759.3 ± 381.8	3275.91 ± 117.9**	3167.7 ± 157.9**	3209.4 ± 183.5**
Central distance travel (cm/10 min)	94.0 ± 13.1	40.9 ± 4.8**	44.4 ± 7.9**	39.6 ± 7.6**
Zone Entries (beam breaks/10 min)	38.5 ± 2.8	24.5 ± 3.5*	24.7 ± 3.4*	25.2 ± 2.4*
Grooming (beam breaks/10 min)	1447.6 ± 46.4	1304.7 ± 34.9	1350.7± 53.5	1420 ± 70.9
Vertical count (beam breaks/10 min)	139.1 ± 7.3	117.4 ± 5.6*	121.8 ± 4.5*	117.9 ± 5.5*
Jump count (beam breaks/10 min)	41.1 ± 2.2	22 ± 4.5**	20.1 ± 4.9**	21.5 ± 3**
**PASSIVE AVOIDANCE**				
Latency Time (s)	294.9 ± 3.5	215.4 ± 23.8*	221.2 ± 30.3*	228.4 ± 17.5*
**MORRIS WATER MAZE**				
Time taken to reach the target quadrant (s)	1.6 ± 0.4	3 ± 0.4*	3.6 ± 0.8*	3.2 ± 0.6*
Time spent in the target quadrant (s)	35.9 ± 2.9	27.6 ± 1.9*	26.3 ± 1.5*	28.4 ± 1.3*
Swim speed (cm/s)	85.8 ± 6.5	65.4 ± 5.4*	56.3 ± 4.7*	59.2 ± 5.9*

Data are the mean ± S.E.M; *p < 0.05, **p < 0.01 vs. CHOW rats.
