# Correction: Micioni Di Bonaventura et al. Brain Alterations in High Fat Diet Induced Obesity: Effects of Tart Cherry Seeds and Juice. *Nutrients* 2020, *12*, 623

**DOI:** 10.3390/nu16111574

**Published:** 2024-05-23

**Authors:** Maria Vittoria Micioni Di Bonaventura, Ilenia Martinelli, Michele Moruzzi, Emanuela Micioni Di Bonaventura, Maria Elena Giusepponi, Carlo Polidori, Giulio Lupidi, Seyed Khosrow Tayebati, Francesco Amenta, Carlo Cifani, Daniele Tomassoni

**Affiliations:** 1 School of Pharmacy, Pharmacology Unit, University of Camerino, via Madonna delle Carceri, 9, 62032 Camerino, Italy; 2 Department of Medicine, University of Leipzig, Liebigstraße 21, 04103 Leipzig, Germany; 3 School of Biosciences and Veterinary Medicine, University of Camerino, via Gentile III da Varano, 62032 Camerino, Italy

In the original publication [1], there were two mistakes in Figure 3 and Figure 7. Unfortunately, in Figure 3, panel E, an error occurred during assembly, with the wrong image used of DJS from our collection, which is now replaced with the correct image. Then, in Figure 7, the beta-actin blots were similar in different brain areas; as a result, we did not realize that there was an error during assembly, with the incorrect blot used in panel A. Figure 7 is now modified to show the correct blot. The corrected figures appear below.

The authors apologize for any inconvenience caused and wish to make clear that the scientific conclusions are unaffected by the corrections herein. These corrections were approved by the Academic Editor, and the original publication has also been updated.

## Figures and Tables

**Figure 3:**
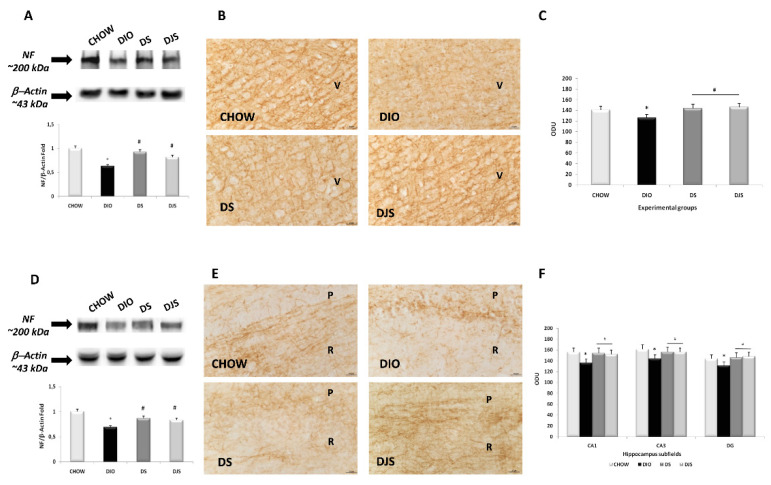
Expression of neurofilament in the different brain areas. Western blot analysis (representative of three different experimental sessions) for samples of the frontal cortex (**A**) and hippocampus (**D**) were probed for NF and β-actin, with corresponding densitometric analysis of the bands. Sections of the frontal cortex (**B**) and CA1 subfield of the hippocampus (**E**) were processed for neurofilament immunohistochemistry, with densitometric analysis of the immunoreaction intensity for the frontal cortex (**C**) and hippocampus (**F**). V, the fifth layer of the frontal cortex; P, pyramidal neurons; R, *stratum radiatum* of the hippocampus. The values are the mean ± S.E.M.; * *p* < 0.05 vs. CHOW rats; ^#^ *p* < 0.05 vs. DIO rats. Calibration bar: 25 μm.

**Figure 7:**
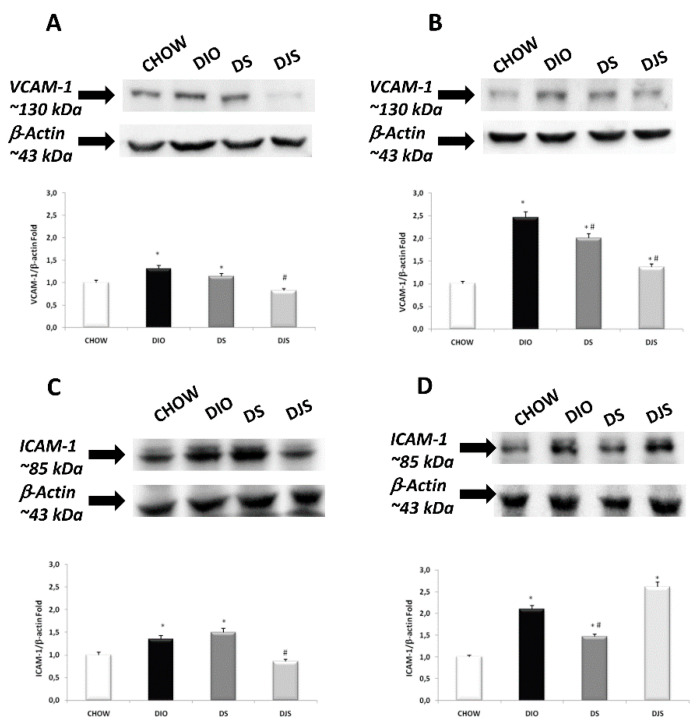
Western blot analysis (representative of three different experimental sessions) for VCAM-1 in the frontal cortex (**A**) and hippocampus (**B**), and for ICAM-1 in the frontal cortex (**C**) and hippocampus (**D**) in the CHOW, DIO, DIO after supplementation with tart cherry seeds (DS), and DIO after supplementation with seeds and tart cherry juice (DJS) groups. Graphs represent the intensity of bands normalized to the band intensity of the reference protein β-actin. The values are the mean ± S.E.M.; * *p* < 0.05 vs. CHOW rats; ^#^ *p* < 0.05 vs. DIO rats.
